# Femoral Component Position Can Affect Patellar Congruency After Medial Unicompartmental Knee Arthroplasty

**DOI:** 10.7759/cureus.102837

**Published:** 2026-02-02

**Authors:** Koichi Nakagawa, Kotaro Yamagishi, Akihiro Moritake, Teruaki Hashimoto, Koji Goto

**Affiliations:** 1 Orthopedic Surgery, Kindai University Hospital, Osakasayama, JPN

**Keywords:** femoral component position, femoral valgus angle, medial unicompartmental knee arthroplasty, patellar congruency, patellofemoral osteoarthritis

## Abstract

Purpose

The aim of this study was to clarify whether femoral component positions can affect the postoperative patellar congruency.

Methods

The preoperative and postoperative radiographic data at 1, 3, 6, and 12 months for all medial unicompartmental knee arthroplasties (41 knees in 35 patients) performed from November 2013 to December 2016 were analyzed. Standardized knee radiographs included standing anteroposterior and Merchant’s views. Component positions were evaluated according to the lateral shift of the medial condyle (LSMC) and femoral valgus angle (FVA). Congruency was evaluated according to the patellar congruence angle (PCA), lateral patellar displacement (LPD), and lateral patellar tilting angle (LPTA). Changes in congruence after the operation and correlations between the component positions and congruence were assessed.

Results

PCAs in 22 knees, LPD in 27, and the LPTAs in 20 were greater at one year compared with the respective preoperative angles. The mean PCA, LPD, and LPTA for these knees were significantly greater at one month. Changes in the two parameters for the component position (LSMC and FVA) and changes in the three parameters for the patellar congruency (PCA, LPD, and LPTA) were significantly positively correlated.

Conclusions

Lateral and distal femoral placement of the femoral component is likely to worsen patellar congruency. The anatomical reconstruction of the medial femoral condyle with an appropriate placement is needed to avoid postoperative deterioration of patellar congruency due to overstuffing of the patellofemoral joint.

## Introduction

Unicompartmental knee arthroplasty (UKA) is becoming an increasingly used alternative to total knee arthroplasty (TKA) [1]. Potential advantages of UKA over TKA include faster recovery time, lower cost, lower rate of serious complications, more favorable functional outcomes, with better range of motion, more normal kinematics, and greater patient satisfaction [2-7].

Despite the potential benefits of UKA, some researchers have expressed concerns about the postoperative progression of osteoarthritis (OA) in the patellofemoral (PF) joint, which causes the need for revision surgery and may substantially limit the effectiveness of the long-term results of UKA [8-10]. The rates of PF OA progression in reported series of the fixed-bearing medial UKA range from 17% to 60% [8,11-14]. Progression has been attributed to the ongoing disease process in the lateral facet of the patella and postoperative impingement of the femoral component against the unresurfaced medial facet. Hernigou et al. reported that the OA in the PF and medial PF impingement were observed in 24% and 35% of knees with UKA, respectively, after >10 years of follow-up. These authors stated that OA in the PF joint after medial UKA caused poorer functional outcomes of the procedure and revision surgery, and that medial impingement, which is noted as a notch of subchondral bony erosion in the axial radiographic view of the patella, could be attributable to anterior placement of the femoral component beyond the transitional zones between the trochlea and the condyles [9]. Berger et al. investigated the frequency of PF complications after medial UKA at 11- to 15-year follow-up and reported that the frequency of PF symptoms increased markedly, affecting 10% of patients after >10 years [8]. They concluded that progressive OA of the PF joint is the primary mode of failure of UKA.

Despite its long history and the renewed acceptance of UKA, uncertainty and concerns about the progression of OA in the PF joint after UKA have continued [15,16]. A recent two-year follow-up study reported that the mean patellar congruency angle (PCA) was significantly larger after medial UKA compared with the preoperative mean PCA [17]. Similarly, other authors have reported that the patellar alignment can worsen after medial UKA [18,19]. However, there are few reports of the factors that can affect patellar alignment after medial UKA, and whether there is an association between the femoral component position and patellar alignment after medial UKA remains unclear.

The purpose of this retrospective observational study was to evaluate how femoral component positioning during medial UKA affects postoperative patellar congruency and PF alignment.

## Materials and methods

This study protocol was fully reviewed and approved by our institutional review board (protocol identification number: 23-087). In this study, the radiographic data of all UKAs performed for cases of medial compartment OA between November 2013 and December 2016 were analyzed. Surgical indications for medial UKA included medial compartment OA of the knee, no significant joint space narrowing in the lateral compartment, an intact anterior cruciate ligament (ACL), correctable varus deformity, and fixed-flexion deformity of <10°. Contraindications included the presence of Kellgren-Lawrence (K-L) [20] grade IV or greater, OA of the lateral compartment, PF joint-related pain symptoms, or inflammatory arthritis. One case of UKA with incomplete radiographic records was excluded due to incomplete radiographic records. Based on these inclusion and exclusion criteria, 41 medial UKAs in 35 patients were enrolled in this study.

The mean age of the patients was 71 years (range: 50-91 years), and 13 knees were in men and 28 knees were in women. Thirty-six knees were diagnosed with primary medial OA and five knees with spontaneous osteonecrosis of the medial femoral condyle. The mean follow-up of the patients was 2.3 years (minimum 1.0 year).

In all procedures, a fixed-bearing UKA (TRIBRID Unicompartmental Knee System, Kyocera Corp, Osaka, Japan) was implanted through a medial mini-skin incision. The operation was performed using the so-called “tibia-cut first and spacer-block technique.” The ACL status was checked to ensure that it was functioning well in each knee. The medial collateral ligament was carefully protected throughout the procedure, and no soft-tissue release was performed along the medial side. Anterior placement of the femoral component beyond the transitional zones between the trochlea and the condyles was carefully avoided [9].

**Figure 1:**
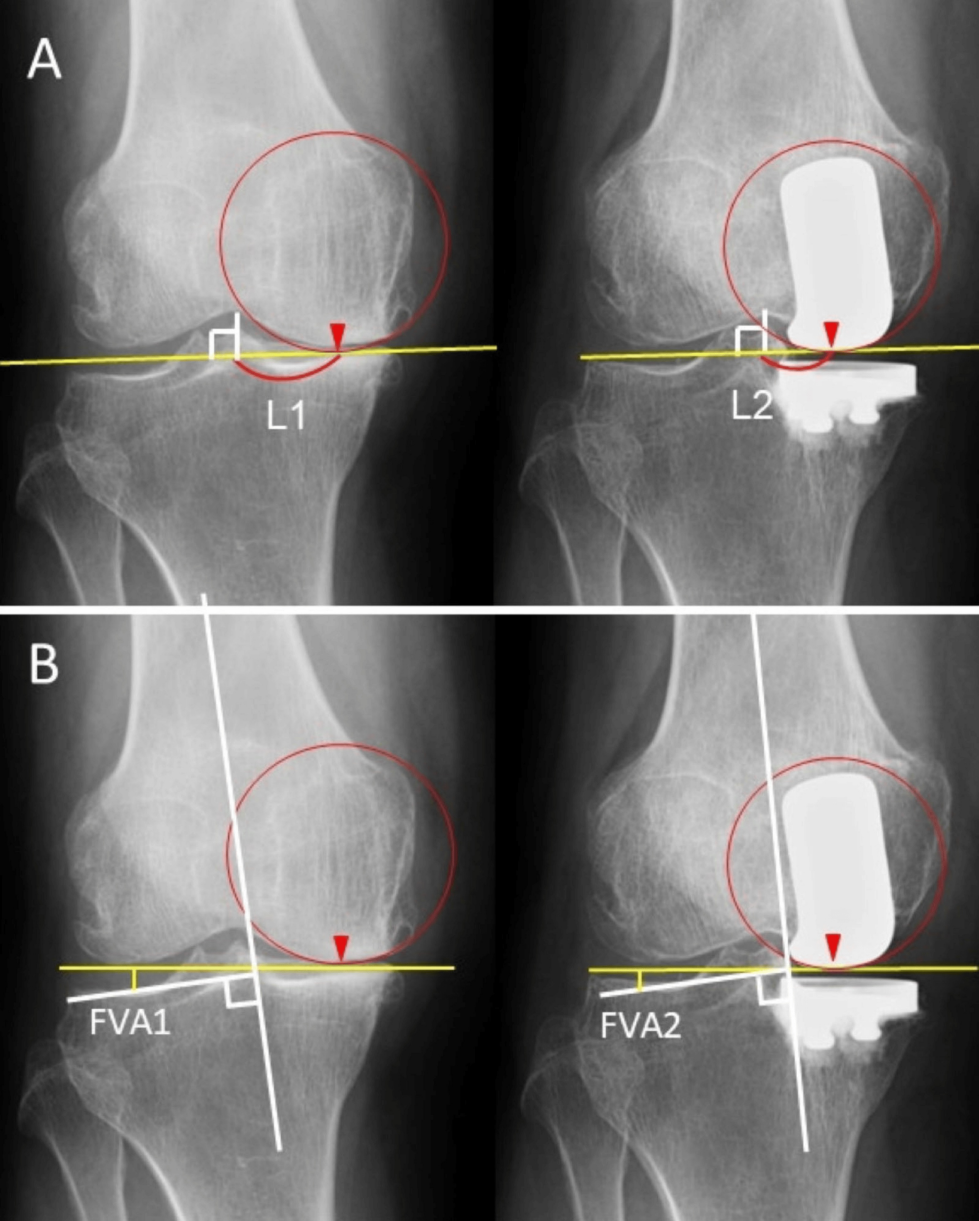
The parameters to evaluate femoral component positions The radiographic images show two parameters to evaluate femoral component positions. The apex of the medial femoral condyle was defined as the lowest point of a circle fitted to the condyle (▼). (A) Lateral shift of the medial condyle is defined as the distance between the apex of the condyle and a line passing through the apex of the intercondylar fossa and perpendicular to the joint line. (B) Femoral valgus angle (FVA) is defined as the angle between the line connecting the medial and lateral femoral condylar apexes and the femoral shaft axis. FVA1 and L1 represent preoperative values, and FVA2 and L2 represent postoperative values.

All patients were followed up at postoperative intervals at 1, 3, 6, and 12 months. Radiographic evaluations that included standing AP radiography and standing Merchant’s view radiography were performed preoperatively and postoperatively using standardized knee radiographs with a measurement scale for distance [21]. The knee alignment in the frontal plane was evaluated by measuring the femorotibial angle (FTA), which is the lateral angle between the axis of the femoral shaft and that of the tibial shaft. The femoral component positions were evaluated using two parameters. The first was a lateral shift of the medial condyle (LSMC), which was defined as the distance between the apex of the medial femoral condyle and a line passing through the apex of the femoral intercondylar fossa and perpendicular to the joint line (Figure 1A)

The second was the femoral valgus angle (FVA), which was defined as the angle between the line connecting the medial and lateral femoral condylar apexes and the femoral shaft axis (Figure 1B).

To determine the position of the apex of the medial condyle on the preoperative and postoperative AP radiographs of the knee, a circle was fit to the preoperative medial femoral condyle on the fully magnified X-ray image and a circle with the same radius was fit to the postoperative medial condyle. The lowest point of the circle was defined as the apex of the condyle.

Patellar congruency was evaluated using three parameters. The first was the PCA, which was defined as the angle between the line from the apex of the trochlear groove bisecting the sulcus angle and the line from the apex of the trochlear groove to the apex of the patella. The second was the lateral patellar displacement (LPD), which was defined as the distance from the medial edge of the patella to a line drawn perpendicular to the anterior condylar line and passing through the anterior-most point on the medial femoral condyle. The third was the lateral patellar tilting angle (LPTA), which was defined as the angle between the line intersecting the widest part of the bony patella and the line passing the anterior surfaces of the femoral condyles tangentially [21-25].

Changes in the three measures of patellar congruency during the postoperative follow-up (1, 3, 6, and 12 months after surgery) were assessed. Pearson’s correlational analyses were performed to examine the relationships between changes (differences from before to 12 months after surgery) in the two measures of femoral condylar shapes and changes (differences from before to 12 months after surgery) in the three measures of patellar congruency.

## Results

The mean preoperative FTA was 181.2° ± 3.2°, and this angle decreased to a mean of 176.6° ± 3.3° after medial UKA (p < 0.0001). The mean preoperative LSMC was 25.6 mm ± 3.2 mm, and this decreased significantly to a mean of 23.3 mm ± 3.7 mm after UKA (p < 0.0001). The mean preoperative and postoperative FVA did not differ significantly (8.6° ± 1.7° and 8.8° ± 2.1°, respectively; p > 0.05) (Table 1).

**Table 1:** Measurement results of each parameter FTA, femoro-tibial angle; FVA, femoral valgus angle; LPD, lateral patellar displacement; LPTA, lateral patellar tilting angle; LSMC, lateral shift of the medial condyle; PCA, patellar congruence angle.

Category	Parameter	Preoperative	Postoperative (one year)	p-value
Knee alignment	FTA (°)	181.2 ± 3.2	176.6 ± 3.3	<0.0001
Component position	LSMC (mm)	25.6 ± 3.2	23.3 ± 3.7	<0.0001
	FVA (°)	8.6 ± 1.7	8.8 ± 2.1	>0.05
Patellar congruency	PCA (°)	-0.39 ± 9.6	3.3 ± 11.4	<0.05
	LPD (mm)	0.3 ± 2.5	1.9 ± 3.9	<0.001
	LPTA (°)	9.7 ± 5.2	10.5 ± 5.3	>0.05

**Figure 2:**
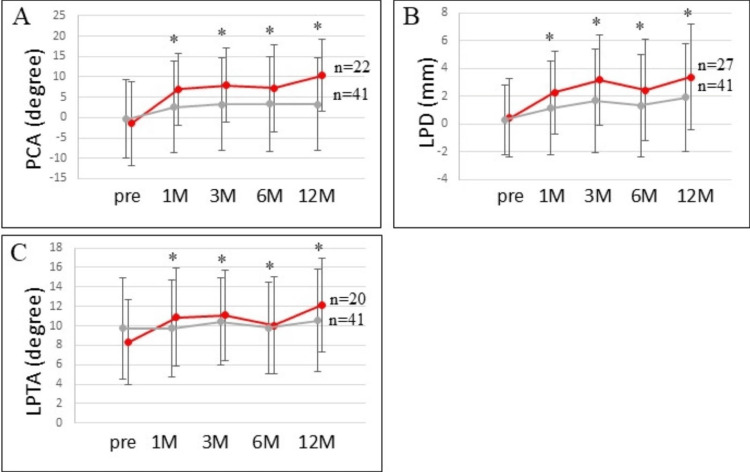
The changes in the three measures of patellar congruency (A) PCA, (B) LPD, and (C) LPTA. The mean PCA in the 22 knees, the mean LPD in the 27 knees, and the mean LPTA in the 20 knees among the total of 41 knees increased significantly at the early postoperative period (red lines and dots). Gray lines and dots indicate the mean values of PCA, LPD, and LPTA among the total of 41 knees. Asterisks indicate statistical significance as compared to the preoperative values (p < 0.05). LPD, lateral patellar displacement; LPTA, lateral patellar tilting angle; PCA, patellar congruence angle.

The mean preoperative PCA was -0.39° ± 9.6°, and the mean PCAs at 1, 3, 6, and 12 months after UKA were 2.6° ± 11.3°, 3.3° ± 11.3°, 3.3° ± 11.7°, and 3.3° ± 11.4°, respectively (Table 1 and Figure 2A).

The mean preoperative LPD was 0.3 mm ± 2.5 mm, and the mean LPD values at 1, 3, 6, and 12 months after UKA were 1.2 mm ± 3.4 mm, 1.7 mm ± 3.7 mm, 1.3 mm ± 3.7 mm, and 1.9 ± 3.9 mm, respectively (Table 1 and Figure 2B). The mean preoperative LPTA was 9.7° ± 5.2°, and the mean LPTAs at 1, 3, 6, and 12 months after UKA were 9.8° ± 5.0°, 10.4° ± 4.5°, 9.8° ± 4.7°, and 10.5° ± 5.3°, respectively (Table 1 and Figure 2C).

The PCA in 22 knees, LPD in 27 knees, and LPTA in 20 knees among the total of 41 knees were worse at the one-year follow-up compared to the respective preoperative alignment. The mean preoperative PCA in these 22 knees was -1.4° ± 10.2°, and the mean PCAs at 1, 3, 6, and 12 months after UKA were 7.0° ± 8.8°, 7.9° ± 9.1°, 7.3° ± 10.7°, and 10.4° ± 8.9°, respectively. The mean PCAs in these knees were significantly larger at 1 and >3 months after surgery compared with before the operation (p < 0.01, Figure 2A). The mean preoperative LPD in these 27 knees was 0.4 mm ± 2.8 mm, and the mean LPD values at 1, 3, 6, and 12 months after UKA were 2.3 mm ± 3.0 mm, 3.2 mm ± 3.2 mm, 2.4 mm ± 3.6 mm, and 3.4 mm ± 3.8 mm, respectively. The mean LPDs in these knees were significantly larger at 1 and >3 months after surgery compared with before the operation (p < 0.01, Figure 2B). The mean preoperative LPTA in these 20 knees was 8.3° ± 4.4°, and the mean LPTAs at 1, 3, 6, and 12 months after UKA were 10.9° ± 5.0°, 11.1° ± 4.6°, 10.1° ± 5.0°, and 12.2° ± 4.8°, respectively. The mean LPTAs in these knees were significantly larger at 1 and >3 months after surgery compared with before the operation (p < 0.01, Figure 2C).

**Figure 3:**
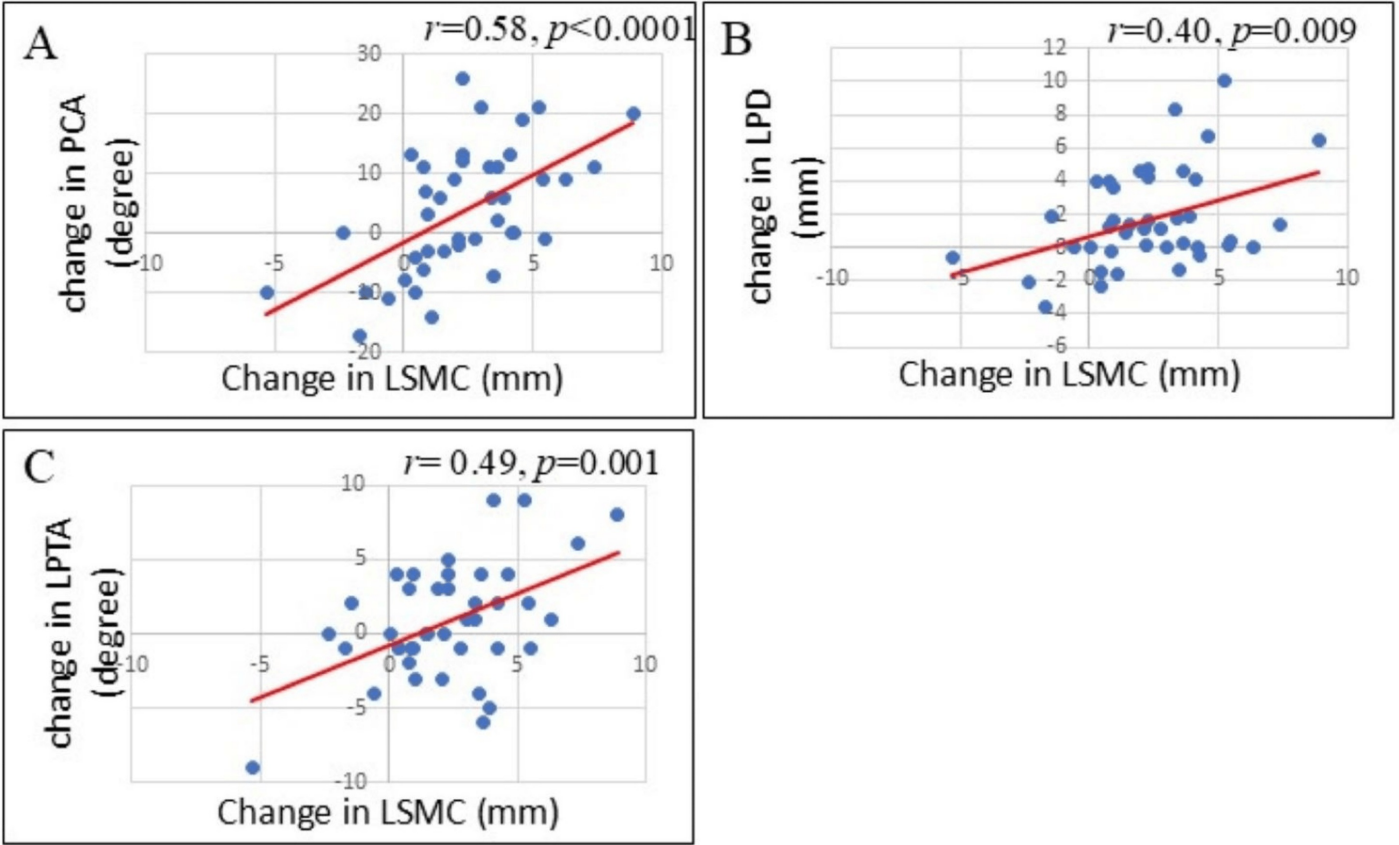
The relationships between the changes in the LSMC and the changes in the three measures of patellar congruency (A) Relationship between LSMC and PCA. (B) Relationship between LSMC and LPD. (C) Relationship between LSMC and LPTA. All three correlations were positive and significant. LPD, lateral patellar displacement; LPTA, lateral patellar tilting angle; LSMC, lateral shift of the medial condyle; PCA, patellar congruence angle.

**Figure 4:**
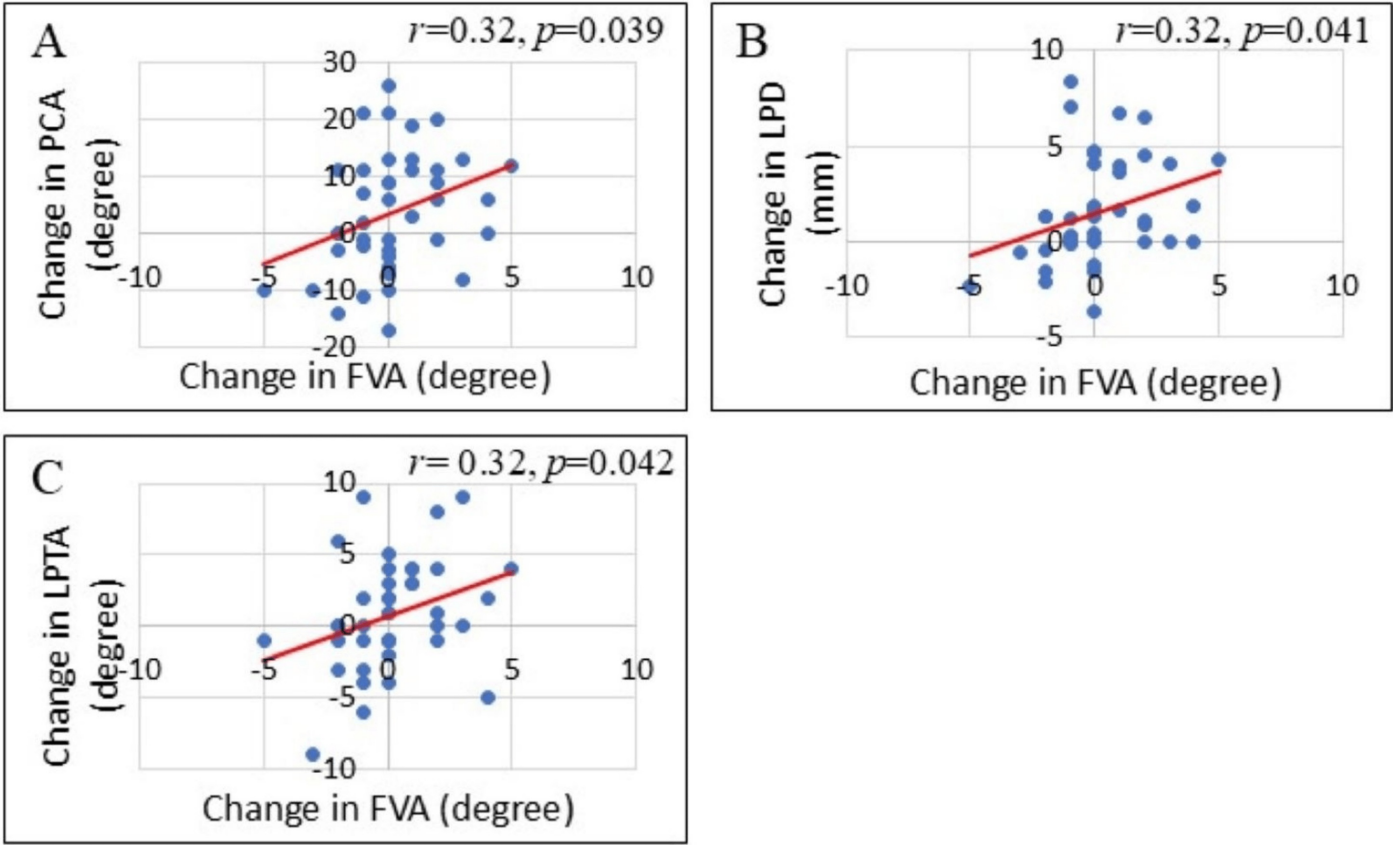
The relationships between changes in the FVA and changes in the three measures of patellar congruency (A) Relationship between FVA and PCA. (B) Relationship between FVA and LPD. (C) Relationship between FVA and LPTA. All three correlations were positive and significant. FVA, femoral valgus angle; LPD, lateral patellar displacement; LPTA, lateral patellar tilting angle; PCA, patellar congruence angle.

Pearson’s correlational analyses were performed to identify associations between changes (differences from before to 12 months after surgery) in the two measures of femoral condylar shapes and changes (differences from before to 12 months after surgery) in the three measures of patellar congruency. The following were significant positive correlations: (a) between changes in LSMC and changes in PCA (r = 0.58, p < 0.0001, Figure 3A), (b) between changes in LSMC and changes in LPD (r = 0.40, p = 0.009, Figure 3B), and (c) between changes in LSMC and changes in LPTA (r = 0.49, p = 0.001, Figure 3C). Pearson’s correlational analysis also showed the following significant positive correlations: (a) between changes in FVA and changes in PCA (r = 0.32, p = 0.039, Figure 4A), (b) between changes in FVA and changes in LPD (r = 0.32, p = 0.041, Figure 4B), and (c) between changes in FVA and changes in LPTA (r = 0.32, p = 0.042, Figure 4C).

## Discussion

To our knowledge, only a few studies have focused on alterations in patellar alignment after UKA. Thein et al. reported that the PCA increased significantly after medial UKA [17], which means that the lateral patellar tilt and shift increased. Similarly, Oh et al. reported an increase in the LPTA after medial UKA, but a decrease in the LPTA after open-wedge high tibial osteotomy [19]. Our results are consistent with these previous observations on the alterations of the patellar alignment.

Theoretically, patellar congruency can worsen after medial UKA, and this worsening may involve progression of ongoing lateral facet OA [3,9], a postoperative decrease in the FTA with an increasing Q angle [18,19], suture insufficiency or looseness of the medial PF ligament (MPFL), and overstuffing of the medial PF joint caused by non-anatomical positioning of the femoral component. Among these effects, we were interested in medial overstuffing of the PF joint because the component positions are under the control of the surgeon.

In terms of the component position, only anterior placement has been reported as a risk factor for PF impingement [3,9]. However, it is possible that mediolateral and distoproximal placement of the femoral component can change the shape of the intercondylar groove. Lateral placement of the femoral component should result in a lateral shift of the medial wall of the groove, and distal placement of the component should result in a distal shift of the medial wall of the groove. These alterations of the patellar groove can cause overstuffing of the medial PF joint when the knee is flexed because both cruciate ligaments are retained in a UKA, which strictly retains the native position of the femur on the tibia. The suture insufficiency of the MPFL and the accelerated progression of lateral facet OA may occur secondary to the overstuffing of the medial PF joint.

The decrease in the FTA after medial UKA may be another explanation for the worsening of patellar congruency. However, a large alignment correction in the lower limb is not performed in UKA because the overcorrection increases the load transmitted through the uninvolved counter-side compartment, which accelerates degeneration [2,26]. Therefore, the increase in the Q angle because of the decreased FTA is considered small, and the effects of changes on patellar congruency may be minimal. In this study, the mean postoperative FTA was 176.6° ± 3.3°, the mean difference in the preoperative to postoperative FTA was 4.6°, and FTA and postoperative congruency did not correlate significantly.

We evaluated the lateral placement of the femoral component using a new radiographic parameter, LSMC, in the AP X-ray film. We chose this measurement as a surrogate indicator of three-dimensional alterations in the medial wall of the patellar groove because the apex of the femoral component can represent the mediolateral component position and is easily compared with the preoperative position. We also evaluated the relationship between the distal placement of the femoral component and the preoperative to postoperative change in the FVA because this angle directly reflects the distal placement of the femoral component and is easily compared from before to after the operation.

In this study, in approximately half of the knees, patellar congruency worsened at the one-year follow-up compared with the preoperative state. In these knees, patellar congruency had significantly worsened in the early postoperative periods. These changes suggest that suture insufficiency or looseness of the MPFL occurs early in the postoperative period, when patients may perform postoperative exercises for range of motion eagerly. If looseness of the MPFL does not occur, deep knee flexion may be limited in patients with malposition of the femoral component because of overstuffing of the medial PF joint. Further progression of the lateral shift and tilt one year after the surgery may include the progression of OA in the lateral facet of the patella, in addition to the effect of looseness of the MPFL.

A statistically significant difference was noted between mean preoperative and postoperative LSMC, which indicates that, on average, the femoral components were placed laterally in this series compared with the original shape of the medial condyle. It is possible that the surgeons tended to avoid polyethylene edge-loading and medial tibial condylar fracture caused by medial placement of the component [27]. In addition, the LSMC correlated significantly with the three measures of patellar congruency, which suggests that the lateral placement of the component is a risk factor for worsening of patellar congruency.

The mean preoperative and postoperative FVA did not differ significantly. This result suggests that surgeons can replicate the original FVA, on average, by resecting the distal part of the medial femoral condyle at the same thickness as the distal part of the femoral component. However, the FVA correlated significantly with the three measures of patellar congruency, which suggests that the distal placement of the component is another risk factor for the worsening of patellar congruency. Distal placement of the component can occur when the bone that is thinner than the component is resected from the distal part of the medial femoral condyle. This technical error may happen if the distal medial condyle experiences eburnation caused by long-lasting OA. That is, the saw blade may be repelled because of the hardness of the subchondral bone and the orientation of the saw blade nearly tangential to the articular surface. Inadequate bone resection from the distal medial condyle or insufficient impaction of the femoral component can result in an increased FVA. Surgeons should therefore pay close attention to avoid both overcorrection of the knee alignment and postoperative patellar incongruency because of the distal placement of the femoral component.

A report on the association between preoperative LPD and poor clinical outcomes following medial UKA is interesting as a relevant study [28]. There might be some patients with the lateral and/or distal femoral component placement in their study in the same way as this study, in whom acceleration of the ongoing lateral facet OA might have resulted in poor outcomes due to anterior knee pain.

Finally, we acknowledge some limitations. First, this study was retrospective and observational, and thus, the evidence level is not high. A prospective cohort study is needed to confirm our results and provide a higher level of evidence. However, after completing this study, we have since started to avoid distal and lateral placement of the femoral component, and for ethical reasons, it is difficult for us to perform a prospective cohort study. The second limitation is that measurements were taken using the AP view of plain X-ray film and not with three-dimensional images, such as CT scans. Measuring the alterations in the medial femoral condyle three-dimensionally would provide further information. However, the LSMC and FVA used in this study may be convenient for the evaluation of the component position because these measurements are easily available in the clinical setting. A third limitation is that the study population was limited to a small number of Japanese patients. Further studies in different and larger populations are needed. The fourth limitation is that clinical outcomes were not evaluated; therefore, the clinical relevance of the radiographic (X-ray) findings remains unclear, and further studies are required.

## Conclusions

Our data suggest that the femoral component position can affect patellar alignment after medial UKA. Other than the anterior placement, the lateral and/or distal placement of the femoral component seems to be a risk factor for worsened patellar congruency. Surgeons should be careful about the mediolateral placement of the component and the appropriate bone resection of the distal part of the condyle for anatomical reconstruction of the medial femoral condyle when performing medial UKA.
